# Facilitators and barriers of intersectoral co-operation to promote healthier and more environmentally friendly behaviour: a qualitative evaluation through focus groups for the INHERIT project

**DOI:** 10.1186/s12889-022-12974-8

**Published:** 2022-03-29

**Authors:** Nina van der Vliet, Lea den Broeder, Maria Romeo-Velilla, Brigit Staatsen, Hanneke Kruize, Bettina Friedrich, A. Jantine Schuit

**Affiliations:** 1 National Institute for Public Health and the Environment, Bilthoven, Netherlands; 2 Tilburg School of Social and Behavioral Sciences, Tilburg University, Tilburg, Netherlands; 3 Achieve, Faculty of Health, Amsterdam University of Applied Sciences, Amsterdam, Netherlands; 4 EuroHealthNet, Brussels, Belgium; 5 University College Leuven-Limburg, Leuven, Belgium; 6 Institute of Child Health, University College London, London, UK

**Keywords:** Intersectoral co-operation, Case studies, COM-B, Behaviour change, Health behaviour, Environmentally friendly behaviour, Equity, Focus groups, Qualitative research

## Abstract

**Background:**

Tackling challenges related to health, environmental sustainability and equity requires many sectors to work together. This “intersectoral co-operation” can pose a challenge on its own. Research commonly focuses on one field or is conducted within one region or country. The aim of this study was to investigate facilitators and barriers regarding intersectoral co-operative behaviour as experienced in twelve distinct case studies in ten European countries. The COM-B behavioural system was applied to investigate which capabilities, opportunities and motivational elements appear necessary for co-operative behaviour.

**Method:**

Twelve focus groups were conducted between October 2018 and March 2019, with a total of 76 participants (policymakers, case study coordinators, governmental institutes and/or non-governmental organisations representing citizens or citizens). Focus groups were organised locally and held in the native language using a common protocol and handbook. One central organisation coordinated the focus groups and analysed the results. Translated data were analysed using deductive thematic analysis, applying previous intersectoral co-operation frameworks and the COM-B behavioural system.

**Results:**

Amongst the main facilitators experienced were having highly motivated partners who find common goals and see mutual benefits, with good personal relationships and trust (Motivation). In addition, having supportive environments that provide opportunities to co-operate in terms of support and resources facilitated co-operation (Opportunity), along with motivated co-operation partners who have long-term visions, create good external visibility and who have clear agreements and clarity on roles from early on (Capability). Barriers included not having necessary and/or structural resources or enough time, and negative attitudes from specific stakeholders.

**Conclusions:**

This study on facilitators and barriers to intersectoral co-operation in ten European countries confirms findings of earlier studies. This study also demonstrates that the COM-B model can serve as a relatively simple tool to understand co-operative behaviour in terms of the capability, opportunity and motivation required amongst co-operation partners from different sectors. Results can support co-operators’ and policymakers’ understanding of necessary elements of intersectoral co-operation. It can help them in developing more successful intersectoral co-operation when dealing with challenges of health, environmental sustainability and equity.

*Coming together is a beginning;*

*keeping together is progress;*

*Working together is success.*

-Edward Everett Hale

Current ways of consuming, moving, and living are often unhealthy and environmentally unsustainable, harming both our health and our planet, thereby endangering future generations. Although people seem willing to adopt a healthier lifestyle that is also more environmentally friendly, many do not succeed in actually doing so. This is partially because most of our behaviour is habitual and influenced by contextual factors: the environments and ultimately the systems in which behaviour takes place. Moreover, these environments differ between population subgroups, creating unequal opportunities and contributing to health inequalities [1]. Changing our lifestyles to become healthier and more environmentally friendly requires not only to be capable and motivated to change, but also to have the opportunity to do so [2]. This ultimately necessitates changing our contexts, environments and systems and the environmental agents that shape these systems [3–5]. Since challenges of public and environmental health, sustainability and equity are interrelated and transcend sectoral boundaries, these so-called change agents also need the opportunity, capability and motivation to co-operate with other agents and sectors. Therefore, understanding and strengthening the behavioural elements that are behind effective intersectoral co-operation is of great value to stakeholders who work towards the integration of knowledge and action of multiple sectors and co-operate intersectorally or across different levels of administration.

## Intersectoral co-operation

Working together with other sectors has been termed differently across studies (e.g., intersectoral action, multisectoral collaboration, or intersectoral collaboration). We use the term ‘intersectoral co-operation’ (IC). Following definitions by Kirch et al. and the WHO, IC entails co-operation between partners from different sectors that makes it possible to take action that is more effective or efficient than taking action by one of the sectors alone [6, 7]. Partners can be from different policy sectors, public and private sectors, different types of institutes, different levels of government, and non-governmental organisations that represent citizens. The goal of IC is to achieve a common understanding on an issue and negotiate and implement mutually agreeable plans. Each co-operation partner brings a distinctive set of assets to the table, which can be usefully combined to solve complex problems.

The importance of working across sectors is emphasised in the field of environmental sustainability. An example being the interlinked Sustainable Development Goals (SDGs: 17 global goals representing a call to action to achieve a better and more sustainable future for all). IC is also part of the health in all policies (HiAP) approach (systematically considering the health implications of decisions of policies across sectors) and whole-of-society or systems approaches (acknowledging the contributing roles played by all relevant stakeholders, including individuals and communities, media and the private sector) [8–11]. The latter approach is also considered necessary when dealing with the wider social determinants of health, widely acknowledged to affect health and health equity [12–14].

## Previous literature

A recent meta-narrative review on intersectoral action beyond health showed a clear rise of publications on IC since 2011 [9]. Although the necessity of co-operation across sectors has been widely acknowledged for some time, in practise, IC is only slowly progressing [14–16]. Literature suggests that the challenging nature of IC contributes to this slow progress. Co-operating partners from different sectors each come from a unique culture of thinking and communication, and they often have distinctive goals and benefits for their own sector. Co-operating partners may fail to find common goals and benefits or lack the commitment to  common goals [7, 12, 17, 18]. Luckily, previous studies have also discovered several facilitating factors, including building and having trusted relationships, formalised and efficient structures and processes to facilitate intersectoral work. Other known facilitating factors include achieving consensus on plans and common goals when initiating the co-operation and believing in the usefulness of co-operating with other sectors [7, 9, 12, 17–19].

## Capability – opportunity – motivation- behaviour

Several theoretical frameworks have been developed and used to understand IC, such as the Bergen Model of Collaborative Functioning [19], the Diagnosis of Sustainable Collaboration model [20], or the Theoretical Model for Reducing Inequalities [12, 21]. Co-operation is a matter of behaviour, hence understanding co-operation between people from different sectors is about understanding (factors influencing) their behaviours. Therefore, we used the COM-B model of behaviour (COM-B; see Box 1) to analyse and report the facilitators and barriers of co-operation [2]. The COM-B has been applied to many types of behaviours, such as healthier and more sustainable diets or physical activity. Moreover, it is applicable to both individual and group behaviour, which makes it a suitable model to study IC group processes [22]. Compared to other theoretical frameworks, the COM-B focuses on understanding the people involved in the co-operation, instead of only focusing on the system in which they operate. Another advantage of using the COM-B lies in the fact that it combined previously developed models and simplified it into one coherent model, facilitating a relatively simple and intuitive way of understanding the conditions needed for IC.

**Table:** 

Box 1. The COM-B model of behaviour [2]. The COM-B model proposes three factors that influence and interact with behaviour (in this case co-operative behaviour):	
• Capability (being able to perform a behaviour, e.g., are partners able to co-operate, what are necessary skills?);	
• Opportunity (having a facilitating social and physical environment that allows for a behaviour, e.g., do partners have the necessary time and resources and social influences?);	
• Motivation (both automatic and reflective brain processes such as intention, attitude, habits, e.g., do partners feel engaged, do they have common values and attitudes?).	

## Aims and contributions to the field

Our research aim was to investigate the perceived facilitators and barriers to intersectoral co-operative behaviours to gain insights into how IC can be organised successfully when working on intersectoral, multi-level challenges of health, environmental sustainability and equity. The outcomes of this study can add to the existing knowledge base in several ways. Most previous studies stem from the field of health science, with only a smallfraction originating from the field of environmental science. In addition, studies are often conducted within a single country or region, frequently focusing on one topic. We included a wide variety of European case studies across a range of topics, that simultaneously address challenges of health, sustainability and/or equity. Hereby, we expand the knowledge base by offering a broader perspective on IC. A variety of study designs have been used to study IC: conceptual studies, document reviews or analysis, questionnaires and semi-structured interviews [9]. A recent study on success factors of intersectoral co-operation used small group discussions to inform thematic coding for subsequent interviews [23]. So far, focus groups appear to be rarely used to study IC processes, hence our study may generate new insights in intersectoral co-operation processes by focusing on the subjective experiences of groups of co-operating stakeholders.

Moreover, to our knowledge, the usefulness of the COM-B to understand facilitators and barriers of IC has not been widely demonstrated. Others have previously applied the COM-B to understand the perception of local policy officials on IC or to understand the role of health brokers who support complex public health problems by facilitating IC [18, 24]. We aim to demonstrate the usefulness of the COM-B in a wide variety of case studies. Our results can support co-operating partners working on a variety of intersectoral topics by providing insights into what aspects to include, develop or strengthen when initiating and maintaining successful IC.

## The INHERIT project

This qualitative study was part of the 4-year EU-funded Intersectoral Health and Environment Research for InnovaTion project (INHERIT) that ran from January 2016 to December 2019. INHERIT aimed to understand how lifestyles and behaviours can be changed in order to promote health, environmental sustainability and equity simultaneously to achieve a ‘triple win’.

## Methods

### Design

For the INHERIT project, several evaluations were conducted on 12 INHERIT case studies that aimed to achieve this ‘triple win’, focusing on its implementation, impacts, cost-benefits and/or intersectoral co-operation [25]. To study IC, we conducted twelve focus groups from October 2018 to March 2019. The focus group methodology is described in detail in a protocol paper [26] and will only be briefly explained in this paper.

### Participants

Twelve focus groups were conducted, one for each case study. This number was set in advance to allow for stringent planning to enable data collection and analysis in the tight timeframe of the project. Focus groups took place in ten different European countries. See Table 1 for the name, country and description of each case study [26]. Each focus group consisted of five to eight participants who were or had been involved in the co-operation process of that specific case study (average of six participants, with a total of 76 participants). Key co-operating partners were selected together with local research teams and case study contact persons. In all focus groups, case study coordinators were present. Ten focus groups included representatives of public administration (the city council or municipality) from various sectors or management/administration levels, such as departments of public affairs, city development, equal opportunities or management. Representatives of citizens were present in five focus groups, and four focus group included researchers from the INHERIT project team that were involved in implementation of specific case studies. This resulted in focus groups existing of various combinations of policymakers, case study coordinators, governmental institutes and/or non-governmental organisations representing citizens or citizens themselves.

**Table 1:** Overview of the 12 case studies, with name, country and short description. Source: [26]

Case study	Country	Short description
Voedseltuin	The Netherlands	A food garden that produces ecologically sustainable vegetables and fruit, working with volunteers with a distance from the job market
Gardening with Green Gyms for Meat Free Monday	United Kingdom	Two sustainable practices combined at a London primary school: meat-free Monday initiative and a Green Gym school garden
GemüseAckerdemie	Germany	Educational program that strengthens the relationship between children and nature, while increasing children’s knowledge of food origins
Ghent en Garde: STOEMP initiative	Belgium	The STOEMP initiative, as part of the Ghent en Garde food policy, is a network that brings good (healthy and sustainable) food initiatives together in the city of Ghent
PROVE	Portugal	A program to create close links between consumers and producers of agricultural products to promote consumption of seasonal fruit and vegetables
Restructuring residential outdoor areas	Sweden	Involving residents to restructure one of the most deprived areas in Stockholm to a more attractive and green outdoor environmental area
Restructuring green space	The Netherlands	Green space neighbourhood park intervention in a low-income urban area in Breda
Sustainable schools in public schools	Spain	Sustainable food in public nursery schools in Madrid, advising parents and training school kitchen personnel to raise awareness in families
Place Standard Tool Latvia	Latvia	Applying the PST to assist professionals and communities in identifying what works well and what needs improvement within a local community, bringing public health, inequalities and environment together in order to create a healthy neighbourhood (Riga)
Place Standard Tool Macedonia	Republic of Macedonia	Applying the PST to assist professionals and communities in identifying what works well and what needs improving within a local community, bringing public health, inequalities and environment together in order to create a healthier neighbourhood (Karposh)
UrbanCyclers	Czech Republic	An urban cycling app to promote sustainable mobility by supporting and motivating self-regulated behavioural change
Eco Inclusion	Germany	A training for refugees to help them save energy in their homes, using a peer-to-peer principle (Pforzheim)

### Central coordination procedures

**Fig. 1:**
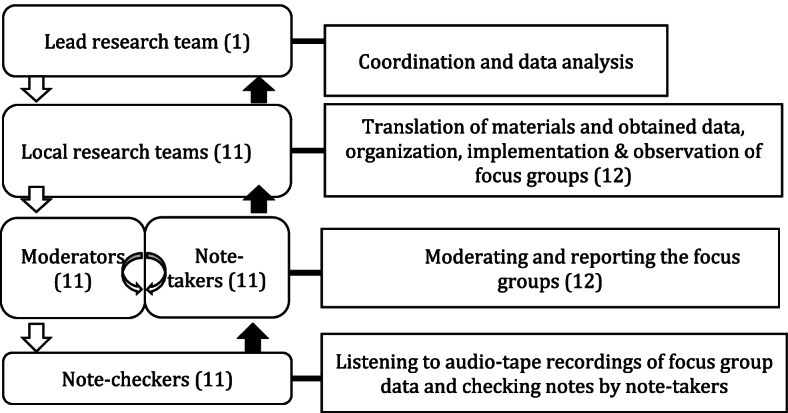
Procedures and roles of focus group process with the number of teams, persons and focus groups between brackets. Source: [26]

This study followed a stepped design with central coordination and data analysis and local implementation and reporting (see Fig. 1 for an overview of the study procedures and roles) [26]. This prevented language and cultural barriers that could have posed an issue because the case studies were taking place in different countries. One lead research team (NvdV, LdB, BS, HK) coordinated the data collection that was carried out by local research teams in the different European countries. A webinar and a handbook were developed including reporting forms and checklists to guide local research teams through the implementation process of each focus group [26]. This included information on core principles of the focus groups and on planning, conducting, note taking and reporting to the lead research team. Local research teams from INHERIT project partners in the respective focus group country conducted and reported the focus groups and translated the reports to English, after which the lead research team analysed data from each focus group. In addition, the lead research team held one online review session with each local research team to check analysis results and to reflect on the focus group.

### Local implementation procedures

The same questions were asked across each focus group. In addition, there was some room for additional questions, allowing for flexibility. Focus groups lasted between 90 and 120 min. All twelve focus groups were guided by a native-speaking moderator or someone with high proficiency in the respective language and supported by a note taker who took notes of the discussions using a standardised form [26]. Participants provided written informed consent before starting the focus groups.

### Appreciative inquiry

Focus groups consisted of four core topics with corresponding questions during the focus group discussions (see Table 2), which were inspired by Appreciative Inquiry, a strength-based approach that directs participants to focus on those aspects of co-operation that have been going well, and how to expand and improve those aspects in future co-operation [27, 28]. In addition, participants were indirectly asked about barriers by asking them what they missed in co-operation and what could be improved, maintaining a solution-based focus towards barriers. Appreciative Inquiry can help people identify those characteristics of the co-operation they want to see more of, in order to create a shared vision of the future and to work towards that future together. The usefulness of Appreciative Inquiry has been demonstrated in interviews and in the development of a coordinated action checklist [28, 29].

**Table 2:** Overview of focus group topics and questions. Source: [26]

Topic (time allocation)	Questions
i. Start and development of the co-operation (±10 min discussion)	“How did the co-operation/project start?” “How did it develop to where it is now?” “What contributed to the co-operation process?”
ii. Core (success) factors of the co-operation (±15 min discussion)	“What are the core factors that made this co-operation happen, that energised and inspired co-operation?” “Describe a peak experience in (intersectoral) co-operation in [case study X], when you felt really engaged and motivated”
iii. Core barriers, challenges, missing in the co-operation (±15 min discussion)	“How could the co-operation have been?” “What would you change if you could change anything in this co-operation? What could it still become?”
iv. Future of the co-operation (±15 min discussion)	“Where do you want to be between now and a certain period, what does this future look like? If your dream is X, what would you want to have accomplished in Y years?” “What are possible options (actions, projects) to reach this and enhance co-operation in the future?”
Wrap up, summary by moderator (±5 min)	“Of all things discussed, what was the most important to you regarding intersectoral co-operation?”

The note-takers used a standardised form to take notes of focus group discussions. Sticky notes were used to collect individual input from the participants on core success factors and future co-operation. This content was then discussed with the whole group. In addition, all focus groups were recorded, after obtaining written permission from participants. The note-taking form was checked and optionally corrected by a second person, who had preferably been present at the focus group as an observer and who used the audio-recordings for reference. Any disagreements between the note taker and the second person were discussed to ensure consensus.

### Data analysis and theoretical framework: COM-B

The lead research team used thematic analysis, which allows finding themes and patterns within and across the data set of the twelve focus groups [30]. Analysis was mainly deductive since we based our theoretical framework and code tree on previous IC literature and the COM-B model for behavioural change [7, 12, 22]. The COM-B model additionally served as a tool to structure IC facilitators and barriers in capability, opportunity and motivation: behavioural preconditions that are needed for behaviour to occur. Emerging themes that did not fit the analytical framework themes were considered to allow for new insights. Further details about the theoretical framework and code tree used for analysis can be found elsewhere [26].

## Results

**Fig. 2:**
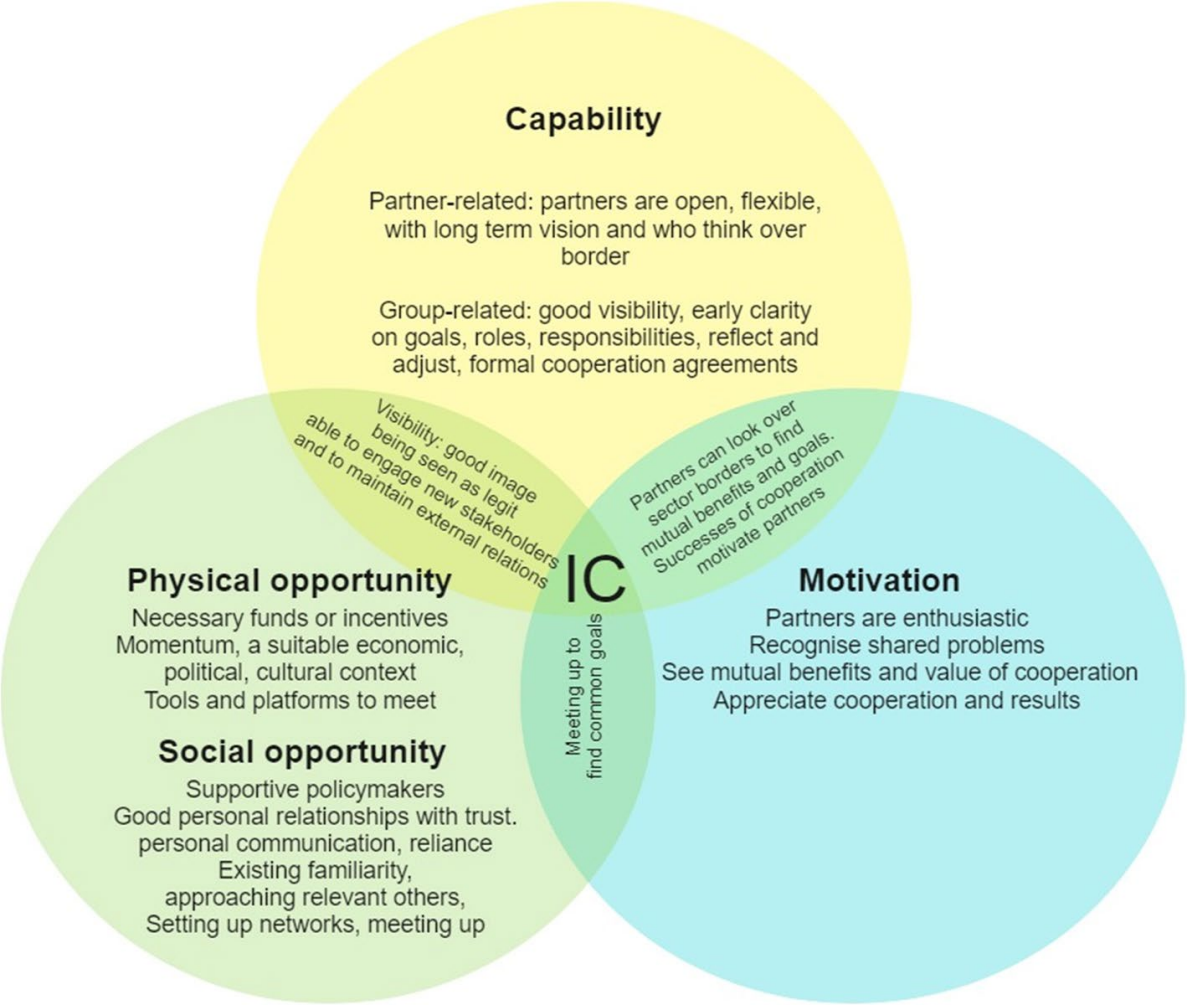
Facilitators for Intersectoral Co-operation (IC) in twelve focus groups categorised in capability, opportunity and motivation from the COM-B model as major themes [2]

In Fig. 2, the results are visualised in the major themes capability, opportunity and motivation and their related facilitator subthemes. As becomes clear from Fig. 2, some of the facilitators are related to more than one aspect at a time. For example, partners must be able to look over sector borders and need to be motivated to find common goals and see mutual benefits of the co-operation with other sectors. Being successful (capability) can result in motivated partners (motivation) and meeting up (social opportunity) can contribute to finding common goals. In Table 3, an overview of the key themes related to facilitators and barriers as discussed in the focus groups is presented. This section elaborates on the themes and is divided into the three major COM-B themes: capability, opportunity, and motivation. For each of these themes, subthemes are organised in facilitators, barriers and future wishes, with an indication of the number of focus groups in which this theme emerged (n/N) and illustrated by examples from the focus group reports. Due to the variety in countries and settings, these reports differed in wording and terminology. Therefore, in this section, we provide paraphrases instead of verbatim quotes.

**Table 3:** Overview of facilitators and barriers of Intersectoral Co-operation categorised in themes capability, opportunity and motivation

Major themes:	Capability	Opportunity	Motivation
Facilitator subthemes	Partner-related: Partners who are open, flexible, with long-term visions Right knowledge and experience and able to think outside sectoral borders A suitable person in leading or guiding position Group-related (group processes): Visibility: positive image, being seen as legitimate, maintaining external relations Working on clarity on goals, roles and responsibility (from the start) Reflecting and adjusting during co-operation Having formal co-operation group or agreements	Physical opportunity (resources): Having necessary funds or financing incentives Momentum, a suitable economic, political or cultural context Tools or online platforms to meet Social opportunity (social influences): Having supportive policy makers Existing familiarity between partners, networks Having good personal relationships between partners (with trust, personal communication, reliance) Meeting up	Motivated partners: Partners who are enthusiastic about co-operating Partners who recognise a shared problem, see mutual benefits and value of co-operation Partners who appreciate the co-operation or results
Barrier subthemes	Insufficient planning, communication and agreement from the start Different working paces or organisational structures between co-operation partners	Physical opportunity: shortages of, or changes in budgets, time, staff Co-operation with public administration or politicians Stakeholders being protective of own work	Negative attitude of parties other than the co-operation partners
Future wishes subthemes	Expanding with more partners or places Clear agreements, increased accountability and responsibility Increased visibility and acknowledgement of initiative and co-operation	More time and structural resources for co-operation and initiative	Boost intersectoral co-operation and willingness to co-operate

### Capability: facilitators

Facilitators related to capability were both partner-related (such as certain knowledge, skills or personalities) and group-related (involving tasks and processes in the co-operating group such as making agreements and coming together to reflect).

#### Partner-related capabilities

In almost all focus groups (10/12), specific capabilities relating to co-operation partners were mentioned as important facilitators. For example, having partners involved who were open to co-operate and other perspectives (7/12), but also partners with a long-term vision, patience, and flexibility allowing them to deal with challenges and the long-term processes of IC (6/12).

—“You can connect those worlds; we have been in those worlds and that is not something every initiative has. It is something, bigger value, knowledge, experience and people who like to puzzle. We can talk with everybody, all layers.”

— participant of Food Garden focus group

In addition, having partners on board with the right knowledge and experience (4/12), who can think broader and beyond their own sectoral borders (3/12) were mentioned. For the case-study Restructuring Residential Areas, this literally meant looking over physical property boundaries and instead looking at a local area as a whole.

*Property owners not only see to their own property boundary; the entire district needs to develop positively according to them.*”

— participant of Restructuring Residential Areas focus group

Moreover, it helped to have a suitable person in a leading or guiding position within the co-operation (6/12), for example someone who keeps everything on track, a process facilitator that maintains an overview and facilitates discussions, or a supportive mayor (outside the project).

“*It helps to have an outside party who can present an objective view. She guided the policy group and discussion as well. She is able to work remotely and has the experience to show for it. She adds some culture to the group: how do you learn to listen to another's opinion.”*

— participant of STOEMP focus group

#### Group-related capabilities

Visibility, having a formal co-operation group or agreements, clarity on goals, roles and responsibility from the start, and reflecting and adjusting during co-operation were important subthemes. Visibility entails having a good image, being seen as reliable and legit and maintaining external relations (5/12). It is also related to being able to motivate and mobilise new partners to allow for expansion of the co-operation (4/12). Participants mentioned the positive reactions and outside interest when they shared their co-operation’s results. It made visible to outside parties what the value of the co-operation was. In addition, being appreciated increased participants’ motivation to continue co-operating.

“*Positive reactions of colleagues in various sectors and their interest in and attention at the presentation of the project in the initial phase, their wish to get introduced to the project and participate in it.*”

— participant of Place Standard Tool Macedonia focus group

*“You must constantly look for alliances, coordinate strengths, teams, alliances… and creating new ones*.”

— participant of Sustainable Food in Nursery Schools focus group

Another group-related facilitator was taking the time to agree on roles and responsibilities and establishing clarity on goals (4/12). In addition, participants mentioned that this should be established not only from the outset of the co-operation, but also during the course of co-operation. For example, providing frameworks on the design of a local green area and making sure that everyone has the same understanding of action points. Partners should take time to “reflect and adjust”: reflect on what has been done and what should be done to move forward (3/12).

*“Proper anchoring - common goals and methods. Everyone pulls in the same direction and knows what issues to work with.*”

— participant of Restructuring Residential Areas focus group

“*Sitting together and regular reflection is very important and should be repeated.”*

— participant of GemüseAckerdemie focus group

In some focus groups, participants mentioned setting up a formal or official co-operation group or agreements between co-operation partners (3/12). For example, an intersectoral co-operation health council working group was started in a municipality and in another case study, there was a board merge between two co-operation partners, allowing for sharing of revenues.

### Capability: barriers

The importance of clarity and communication also became clear from discussions on barriers. Participants wished they would have spent more time on (beforehand) planning and agreements and on more communication from the start (5/12). Another barrier was different working paces or organisational structure of co-operation partners, for example between a more silo-based municipality and intersectorally organised small social organisations (3/12).

*“Maybe we should have linked up more at the start. […] Would have been great to have more time to plan together with P8 [another focus group participant] … that’s really helpful.*”

— participant of Green Gyms and Meatless Mondays focus group

“*Hybridity [with funds from public, private and collective sources] was already a known concept. Now it sounds like a sort of disease, but we are really working on aspects of health and work. From the perspective of the government that is organised differently, we diverge from the norm.*”

— participant of Food Garden focus group

### Capability: future wishes

The most frequently discussed future plans and wishes involved growth (9/12) of the initiative and co-operation. For example, by expanding to other places (such as other nursery schools in the Sustainable Food in Nursery Schools, to other schools’ curricula in the Green Gyms and Meatless Monday), or including new partners such as local businesses (Eco Inclusion) or marketplaces (PROVE). To allow for this growth, participants in the Food Garden case study suggested they could be given a pilot or experiment status.

“*Say, if we would get five years to realise our ideal in co-operation with the municipality, which would also get the time and involve entrepreneurs: the chain idea could move forward […] You would get a bit more time and support, that would be a top story, at both sides, and you can learn from it as well*.”

— participant of Food Garden focus group

Multiple focus groups expressed wishes for clear agreements and increased accountability and responsibility among all partners (5/12):

“*It is also a matter of responsibility, the municipality has laid out and financed and to put it boldly, stops there. Who is then the owner to put 2.0 on the map? If nobody does anything, nothing happens. Somebody must get up and mobilise and brings parties together. If you do not have someone with time and space, then it just does not happen.*”

—participant of Restructuring Green Space focus group

In addition, there was a wish to increase the visibility and acknowledgement of the conducted work (6/12). Suggested ways to achieve this included making the co-operation more formal or official, and by demonstrating results or increasing brand awareness.

*“The municipality mayor should emphasise that the results and recommendations from analysis with the Place Standard Tool will be incorporated in a future programme of the Municipality. Thus, co-operation will be understood as more formal and obligatory*.”

—participant of Place Standard Tool Macedonia focus group

*“I want to increase visibility. To have the STOEMP label, or brand, appear in even more activities. Not just within specific organisations or services, but to really have it come to life.”*

— participant of STOEMP focus group

### Opportunity: facilitators

Opportunity consists of physical opportunities (e.g., from resources such as time and budget or facilitating tools and platforms) and social opportunities (e.g., social influences such as supportive external stakeholders and good personal relationships). Subthemes related to physical opportunity were having the necessary funds or financial incentives to co-operate (4/12), and a suitable economic, political or cultural context (3/12). In the case study PROVE, the surpluses of farmers’ crops made them co-operate to set up a connection between farmers and consumers.

*“At that time, the first thing we did was a diagnosis. To understand the state of farming, how was the local production? And what we found was: our farmers had production, but they didn’t have a way to drain their production.”*

— participant of PROVE focus group

Momentum, or the right timing, was mentioned as a facilitating moment where several circumstances came into effect at once, which got things going (3/12). The economic or political context can also be a trigger, such as the arrival of many refugees for the case study Eco Inclusion in which refugees are taught how to live energy efficiently. For others, political triggers supported setting up co-operation, such as the Milan Food Policy Act which gave rise to the case study of Sustainable Food in Nursery Schools.

A tool or an online platform was seen as facilitating IC by bringing together partners or communities (4/12). In the case of PROVE, an online platform brought promoters, consumers and farmers in contact with each other, and in the case of Sustainable Food in Nursery Schools, kitchen and school staff could share experiences in an online learning community.

*“Exchange spaces […] and [online] learning communities, sharing is very important, to solve doubts and is related to what we spoke about of creating a community.*”

— participant of Sustainable Food in Nursery Schools focus group

*“This tool shows well that human health, well-being and quality of life, health depends on various aspects.”*

— participant of the Place Standard Tool Riga focus group

Subthemes related to social opportunity included having supportive policy makers (i.e., a local mayor or municipality) (6/12), and this was also experienced as a trigger for project initiation (5/12). For example, municipalities facilitated co-operation between different organisations within the area (Food Garden) or provided frameworks for green space design (Restructuring Green Space). In four focus groups, it was mentioned that interest and support from researchers from the INHERIT project was the very reason why the respective co-operation were initiated or expanded: these case studies were piloted for INHERIT and this triggered partners to start co-operating with each other.

Having existing networks or reaching out to previous contacts were experienced as facilitating the initiation of co-operation (5/12). For example, partners worked in each other’s fields before, or worked together in previous projects. Knowing each other and being familiar with each other facilitated development of the co-operation. Previously having worked together also contributed to having personal relationships. These personal relationships in which partners could trust, communicate personally and rely on each other were experienced as facilitating (5/12).

*“I already knew a lot of players, P1 and me are both field workers”. P1: “You have transferred to the other side”. P2:” I first worked at the municipality and now I have transferred to the field. I already knew a lot of players in the city, and along the way we ended up talking.”*

— participant of Food Garden focus group

*“Through reliable project partners, whom you can trust, you get committed to it, so that problems could also be overcome.*”

— participant of Eco Inclusion focus group

Besides having a network of co-operation partners and being able to share experiences, meeting up with co-operation partners was mentioned in many focus groups as being a peak experience and facilitator of co-operation (8/12). Participants seemed to find these meetings (varying from national events to small work group meetings) particularly motivating. It contributed to developing plans and facilitated the development of personal relationships and action. Being able to physically get together to talk and plan for actions helped, for example by organising a kick-off meeting (GemüseAckerdemie) (5/12). These meetings were experienced as supporting clarification and finding common goals and becoming enthusiastic. The process of finding common goals was mentioned in four focus group. Two working groups actually merged when they discovered they were working towards the same goals (STOEMP).

*“Meeting partners has been great, because it materialises from an idea to something that can happen.”*

— participant of Green Gyms and Meatless Mondays focus group

*“‘Thinking about a shared vision and goals together brings the network closer together.*”

— participant of the STOEMP focus group

### Opportunity: barriers

Most of the discussed IC barriers in the focus groups were related to physical opportunity: the extent to which budgets, time and staff for co-operation were available was seen as essential to set up, develop and expand co-operation (6/12). When these elements were not sufficiently present or had changed, opportunities to co-operate, growth and continuity of the case study were negatively affected.

“*We have low amounts of staff. These changes [making nursery school canteens more sustainable] need workforce, and we often lack it.”*

— participant of Sustainable Food in Nursery Schools focus group

Co-operation with public administration or politicians was considered to be a difficult activity in some focus groups (6/12). This could be due to a political agenda or sector-based organisation of the local municipality. A smaller amount of focus groups discussed legislative barriers, such as a difficulty to receive permits and structural contracts for the garden in the Food Garden case study.

In a few focus groups (3/12) participants mentioned another barrier, namely other parties being protective of their own work, or limiting co-operation opportunities.

*“What I've got to know about this world, there are many organisations that are heading to the same goal, but every organisation protects their things a little bit.”*

— participant of Urban Cyclers focus group

### Opportunity: future wishes

Participants expressed the need to involve, reinforce and motivate co-operation with (political) stakeholders (4/12), for example by meeting up. In addition, participants wished for more time and structural resources (5/12). Food Garden participants wished for more integral financing (having a budget for initiatives with an intersectoral nature) instead of many different small subsidies from different sources.

“*One third [of the financing was] collective (social capital, work, inhabitants), one third public, one third private/market finance. A hybrid, integral business model. Partly from the market and partly collective. Now you often are one or the other, and it almost does not exist that you are all three at once. That is my mission. If it succeeds, you can easily make agreements. Then the municipality would say, I participate for one third with that piece. Now it is seen as a whole, and you have to categorise.*”

— participant of Food Garden focus group

### Motivation: facilitators

Motivation entails both ‘reflective’ processes, such as evaluations and beliefs, and ‘automatic’ processes, such as needs, desires and emotions. Generally, subthemes were related to having highly motivated co-operating partners, who had common goals and saw mutual benefits.

Participants from almost all focus groups (10/12) expressed a high motivation to co-operate and make the initiative a success. They were enthusiastic and felt like co-owners of the initiative. In five focus groups, it was discussed that the co-operation was initiated because partners recognised a common problem and the need to tackle the problem together. For example, in the case of Restructuring Green Space, multiple parties were worried about a local deprived neighbourhood in need of development. In addition, almost all focus groups (10/12) mentioned that having and seeing mutual benefits, having shared goals (and taking the time to find these goals), or seeing the value or necessity of co-operation facilitated IC (6/12). For Restructuring Residential Areas and Restructuring Green Space, the common interest that brought partners together was to create safe areas with better quality, which would benefit all co-operation partners. For 3 out of 12 focus groups, having the same goals in terms of benefits for the target group (children) was a clear facilitator.

*“It's exceptional: we all feel like co-owners of the project. Everyone feels involved, despite the fact that we all came in at different times.”*

— participant of STOEMP focus group

*“It is about the same mind-setting. If we did not have the same foundation (both value and practical basis), co-operation would not work so well. It's good for everyone, we all want more cyclists.*”

— participant of Urban Cyclers focus group

Participants mentioned that they appreciated the results of their co-operation (6/12): experiencing good results and seeing success due to the co-operation motivated them to continue co-operating (6/12 focus groups). In many focus groups (9/12), the appreciation of the co-operation itself or the co-operation partners was expressed, for example by noticing enthusiasm, willingness or support from partners.

“*We appreciate that the co-operation is bilateral and supportive.*”

— participant of Place Standard Tool Riga focus group

### Motivation: barriers

Participants sometimes encountered negative attitudes towards the initiative by those outside the co-operation (2/12). For example, directors from the social sector initially looked upon the Food Garden as “*a bunch of cowboys who were working on low hanging fruits*”, but after demonstrating the value of the initiative to the city, they were appreciated. Participants experienced this as a barrier to the development of the co-operation and the initiative.

### Motivation: future wishes

Motivational future wishes were related to increasing willingness to co-operate (among co-operation partners and potential partners) and to deepen their co-operation. In some focus groups, participants expressed a wish to boost IC and eagerness to co-operate, and to make sure the co-operation would be maintained and strengthened in the future (4/12).

*“We need to make sure sustainability becomes a reflex in all health-related matters.”*

— participant of STOEMP focus group

## Discussion

Intersectoral co-operation is considered to be a necessary element of dealing with today’s interlinked challenges of public health, environment and equity [8–14]. However, co-operation beyond sectoral borders is not an easy task. Therefore, knowing the factors that facilitate or hinder IC can support partners to improve or develop their co-operation processes and structures. This qualitative study explored facilitators and barriers in co-operative behaviour, as experienced by co-operating partners in twelve case studies from ten European countries as part of the INHERIT project. Case studies were diverse and aimed to promote both health, environmental sustainability and equity through behavioural or lifestyle change. Despite the diversity of the case studies, we found several common facilitators and barriers.

### Most important facilitators and barriers

For a quick overview of the most important facilitators and barriers, we refer to Fig. 2, in which the major themes and subthemes of capability, opportunity and motivation have been visualised. In addition, Table 3 shows facilitators, barriers and future wishes subthemes, again using capability, opportunity and motivation as the three major themes of IC. In the following sections, findings will be discussed and compared to earlier research.

#### Capability

Capability related facilitators that were mentioned frequently included various aspects of engaging the ‘right people’: people who were willing and open towards co-operation and other perspectives, had long-term vision, patience, and flexibility. A suitable leader or guide of co-operation, being visible (having a good image and being seen as reliable and legit) and being able to expand the co-operation by mobilising and including new partners was seen as important. Moreover, deemed important were having (formal) agreement on roles and responsibilities and being clear on the goals of co-operation from the start, with opportunities to reflect and adjust along the way. Previous research found similar facilitators. For example, others have found that leadership that inspires trust, confidence and inclusiveness facilitated IC, as well as monitoring how communication is received and adjust if necessary [19, 31]. In addition, the Coordinated Action Checklist (a tool to facilitate and evaluate community health promotion) includes having suitable partners who have common goals and agreements, communicate and can mobilise others [29]. A part of the Coordinated Action Checklist was dealing with conflicts in a constructive way, but our participants did not mention such conflicts within the co-operation. This may be because our participants did not experience such conflicts, but it could also be due to social desirability or our Appreciated Inquiry approach, which focused more on success factors and future wishes than barriers.

#### Motivation

Motivation seemed to be the most often mentioned major theme with regard to facilitators. Partners stated that they were highly motivated to co-operate, and they emphasised the importance of recognising a shared problem, finding mutual benefits, and having common goals, as well as acknowledging the necessity of the co-operation as facilitating IC. This confirms findings by two large reviews [9, 19] and a recent large survey about good health-promotion interventions that exemplified effective IC, in which ‘*a shared vision of the problem to be addressed*’ and ‘*a win-win for partners in the collaboration*’ were among the most frequently named key success factors [23]. Barriers related to motivation were identified less often in the current study and experienced barriers were often related to people outside the co-operation (e.g., with a negative attitude towards the co-operation).

#### Opportunity

Opportunities that facilitated intersectoral co-operation were having necessary funding, financial incentives, time and/or staff and having the right economic, political, or cultural context such as supportive policymakers. Earlier studies found that a lack of resources or political commitment can be important barriers, and our study confirms this. Not having structural resources or enough time to set up a co-operation were identified as barriers, sometimes related to not having supportive policymakers or an enabling public administration [7, 17, 19, 32]. Social opportunities experienced by our participants as particularly valuable were having personal relationships with trust and reliance, having the right network, sharing experiences, and meeting up with co-operation partners. These findings are also in line with earlier literature, in which the importance of communication, trust and good relationships between co-operating partners was reported [9, 19, 23].

#### Future wishes

Participants indicated they wanted to boost the co-operation and make sure that all involved partners shared this wish. They wished to grow in terms of partners or other places, as well as increased visibility and acknowledgements from external stakeholders. Also, they wished for clear agreements and responsibility. To accomplish this, more time and structural resources were considered necessary.

### New insights in IC from this study

In general, identified facilitators and barriers to IC are comparable to those found in earlier research. Graham et al. (2018) stated before that there is a knowledge base on what works already [33]. However, actually implementing what works to develop effective IC still appears to be a challenge. It has already been stated that the diversity and complexity of theory is a potential reason for the limited use of theory in intervention design and evaluation [2, 34]. The simplicity of the COM-B categories can promote understanding of collaborators and policymakers of which elements they need to include or improve for more successful co-operation. What skills do partners need, what tasks are essential to organise from the beginning and during co-operation? Are partners motivated, do they recognise common goals and see the added value of the co-operation? Are there enough meeting opportunities to realise this? Are necessary resources available, and do economic or political environments provide the right opportunities to co-operate?

Previous studies have used a wide variety of frameworks to structure their findings on IC [29, 35–37]. To our knowledge, only two have used the COM-B to understand facilitators and barriers of IC that were studied using individual interviews. Hendriks et al. interviewed policy officials in two small Dutch municipalities and van Rinsum et al. interviewed professionals from various backgrounds [18, 24]. Our study adds to the limited amount of research using the COM-B model to gain insights into IC and represent findings in an easy-to-understand manner.

### Strengths and limitations

Previous research has often been conducted in one field (e.g., nutrition) or country, mostly originating from the field of public health. In addition, previous research often used other study methods, such as interviews. We conducted cross-country focus groups on intersectoral co-operation among a wide variety of case studies. Results may therefore be better suited for generalisation beyond the public health field and be applied to other fields such as environmental sustainability. In addition, focus groups allow for discussions between co-operating partners and may result in a fuller impression of IC processes. Experiences shared by an individual participant can immediately be responded to with (dis)agreeance or additional experiences. Moreover, case studies were held throughout Europe, allowing for emerging themes that were cross-cultural. As mentioned before in a review on IC by Corbin et al. (2018) and a recent review by Mondal et al. (2021), a language bias in literature on IC may exist because often only studies published in English are included in reviews. This may result in a bias towards experiences in countries where people master the English language [9, 19]. Our stepped approach allowed for conducting focus groups in different countries, in native languages, therefore we were able to overcome this language bias and compare findings across countries.

#### Differences in implementation and reporting between focus groups

The stepped approach with local native-language data collection and central data analysis allowed for a resource-efficient method that suited the project budgets and timeline. However, even though focus groups were instructed with the same materials providing detailed instructions, there were some differences in implementation and reporting between focus groups. A minority held shorter discussions on certain questions or reported in less detail, which may have resulted in less rich data for these focus groups. However, themes emerging from these focus groups were similar to the themes in the focus groups with more extensive reporting. The number of focus groups was set in advance and focus groups were conducted in parallel, therefore sampling might have ended before data saturation. However, the high number of focus groups and the observation that no new large themes emerged in later focus groups suggest data saturation may have already occurred [38, 39].

#### Translation

There are some other limitations to the study design, which will only be briefly discussed here as they were discussed in the protocol paper in more detail [26]. The first limitation may be that the researcher conducting data analysis was not present at all focus groups and did not take notes due to language barriers. Focus groups were held in the native language, requiring translation and risking losing some of the richness of data in the process. However, conducting focus groups exclusively in English instead of in the native spoken language would have limited access for certain co-operating partners to the focus groups. Participating in a language foreign to the native language could have negatively influenced the quality of data. To partially overcome misrepresentation of data that could arise by this design, the focus group reports were checked by native speakers present at the focus groups.

#### Verbatim transcripts

We did not use verbatim transcripts. Focus group discussions were captured by note-taking, discussed with a second person and checked with the audio-recordings of the focus groups discussions as reference. It has been argued that when using thematic analysis to find common themes, case verbatim transcripts are not always necessary [40, 41]. One study found a high consistency in number and content of themes in the interview data using both verbatim transcription and our method (termed *scribing)* [42].

#### Social desirability

A potential disadvantage of using focus groups instead of individual interviews is the risk of social desirability: participants may have been reluctant to discuss struggles or negative views about other (present) participants. In addition, there are cross-cultural differences, including some cultures where being more direct is more common than in other cultures. In countries where it is less common to be openly critical, this may have been amplified by using Appreciative Inquiry. On the other hand, it may have provided a safer mode of discussing issues as points of improvements in an action-focused way. A common criticism of Appreciative Inquiry is that it prevents discussion of negative aspects, but our participants mentioned they appreciated reflecting on the co-operation together including on which aspects they wanted to strengthen or develop in the future. In addition, we explicitly asked participants to reflect what could be improved (and how), and what participants missed in the co-operation. This allowed for discussions in which barriers could still be expressed in a constructive way including potential solutions.

### Recommendations for future research

This study demonstrates that the use of COM-B makes it relatively simple and easy to understand facilitators and barriers that influence successful IC according to participants’ experiences. Facilitators identified here could be used to develop a guideline for IC in projects that aim to promote health, environmental sustainability and equity. Future research could investigate whether this simpler presentation supports those involved in setting up, developing, improving or facilitating IC.

## Conclusions

Intersectoral co-operation is widely considered to be a necessary ingredient in dealing with the interlinked challenges of health, environmental sustainability, and equity. This qualitative study explored facilitators and barriers of co-operative behaviour as experienced by co-operating partners in twelve diverse case studies throughout Europe. Among the main facilitators experienced were having highly motivated partners who find common goals and see mutual benefits, with good personal relationships and trust. In addition, having supportive environments that provide opportunities to co-operate in terms of support and resources, together with co-operation partners who have long-term visions, create good external visibility and who have clear agreements and clarity on roles from early on. This was a cross-cultural project, suggesting that facilitators that were frequently mentioned are not culturally specific to one European country. Most identified facilitators and barriers to IC are comparable to earlier research. We add to this knowledge base by demonstrating how the COM-B can serve as a relatively simple tool to understand co-operative behaviour in terms of the capability, opportunity and motivation required. This can promote understanding among coordinators and policymakers of what elements they need to include or improve for successful intersectoral co-operation. This understanding can ultimately contribute to tackling challenges of health, environmental sustainability, and equity.

## Data Availability

Materials can be downloaded from the protocol paper [26]. The data analysed during the current study are available on reasonable request from the corresponding author [NV]. The data are not publicly available due to research participant privacy restrictions.

## Abbreviations

COM-b: Capability Opportunity Motivation-Behaviour model by Michie et al. (2011)
IC: Intersectoral Co-operation
INHERIT: INtersectoral Health and Environment Research for InnovaTion
SDG: Sustainable Development Goal
